# Cohesinopathies of a Feather Flock Together

**DOI:** 10.1371/journal.pgen.1004036

**Published:** 2013-12-19

**Authors:** Robert V. Skibbens, Jennifer M. Colquhoun, Megan J. Green, Cody A. Molnar, Danielle N. Sin, Brian J. Sullivan, Eden E. Tanzosh

**Affiliations:** 1 Department of Biological Sciences, Lehigh University, Bethlehem, Pennsylvania, United States of America; 2 Merck, Sharp & Dohme, West Point, Pennsylvania, United States of America; 3 Janssen R&D, LLC, Raritan, New Jersey, United States of America; University of Pennsylvania, United States of America

## Abstract

Roberts Syndrome (RBS) and Cornelia de Lange Syndrome (CdLS) are severe developmental maladies that present with nearly an identical suite of multi-spectrum birth defects. Not surprisingly, RBS and CdLS arise from mutations within a single pathway—here involving cohesion. Sister chromatid tethering reactions that comprise cohesion are required for high fidelity chromosome segregation, but cohesin tethers also regulate gene transcription, promote DNA repair, and impact DNA replication. Currently, RBS is thought to arise from elevated levels of apoptosis, mitotic failure, and limited progenitor cell proliferation, while CdLS is thought to arise, instead, from transcription dysregulation. Here, we review new information that implicates RBS gene mutations in altered transcription profiles. We propose that cohesin-dependent transcription dysregulation may extend to other developmental maladies; the diagnoses of which are complicated through multi-functional proteins that manifest a sliding scale of diverse and severe phenotypes. We further review evidence that cohesinopathies are more common than currently posited.

## Introduction

### A shared genetic basis of developmental abnormalities

**Figure 1:**
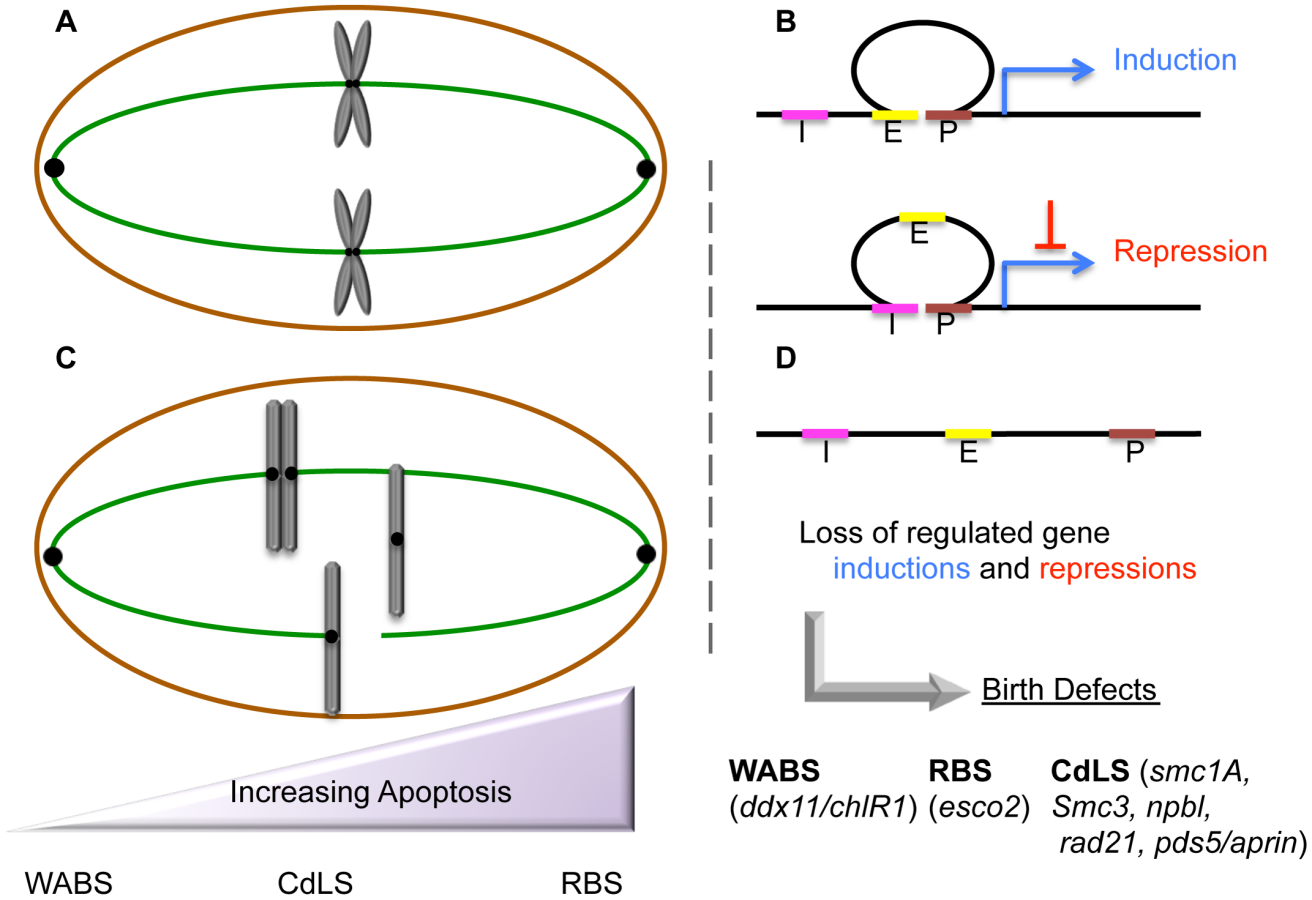
Etiologic and peripheral phenotypes of cohesinopathies. (A) Cohesins maintain sister chromatid tethering required for normal mitosis (chromosomes in gray, microtubules in green) and also (B) stabilize chromatin loops through which developmental transcription programs of gene inductions and repressions are deployed (E = Enhancer in yellow, P = Promoter in brown, I = Insulator in pink, Blue arrow = Transcription). Additional roles for cohesin as boundary elements that demarcate chromatin domains and terminate transcription are not shown. (C) Cohesion mutations exhibit a sliding scale (purple) of phenotypic manifestations that may include chromosome mis-segregation/aneuploidy, chromosome condensation defects, HR/PCS, apoptosis, and genotoxic sensitivities—phenotypes that fail to correlate with genotype. (D) RBS and CdLS cohesion mutations dysregulate transcription profiles, which we speculate produces the developmental defects in all cohesinopathies (RBS, CdLS, and WABS mutated genes shown in lower case). We hypothesize that developmental maladies such as Treacher Collins Syndrome (TCS), Diamond Blackfan Anemia (DBA), and Nijmegan Breakage Syndrome (NBS) are similarly based on transcription dysregulation.

Roberts Syndrome/SC-Phocomelia (RBS) and Cornelia de Lange Syndrome (CdLS) are severe multi-spectrum developmental disorders. Patients afflicted with either RBS or CdLS present with nearly identical phenotypes that include acute long-bone growth failure (near-absence of extremities positions hands and/or feet close to the body), mental retardation, craniofacial malformation, and perturbations of heart, kidney, genital, and gastrointestinal development (Table 1). Consistent with this similar suite of phenotypes, both RBS and CdLS arise from mutations within a single pathway. Mutations in *ESCO2* produce RBS [1]–[3]. ESCO2 is a member of a highly conserved acetyltransferase family (Eco1/Ctf7 in budding yeast, ESCO2/EFO2 ESCO1/EFO1 in humans) that is essential for sister chromatid tethering reactions (termed cohesion) and high fidelity chromosome segregation [4]. To date, the only known essential substrate of ESCO2 is the cohesin protein SMC3. Cohesin complex (which also contains SMC1A, MCD1/SCC1/RAD21, and Irr1/Scc3/SA1,2/STAG1,2) binding to DNA requires a deposition complex that contains SCC2/NIPBL and SCC4/MAU2 [4]. Mutations within cohesin subunits (SMC1A, SMC3, and RAD21) and cohesin auxiliary factors (NIPBL, HDAC8, and cohesin-associated PDS5/APRIN) give rise to CdLS [5]–[12]. Despite the common manifestations and genetic basis of RBS and CdLS, these developmental abnormalities are thought to arise through different responses to cohesion factor mutations: RBS through elevated levels of apoptosis and limited progenitor cell proliferation, and CdLS through transcription dysregulation. The transcription-based CdLS model is supported by findings that CdLS cells do not exhibit elevated levels of apoptosis or mitotic failure, and that chromatin-bound cohesins not only participate in sister chromatid tethering, but also i) participate in boundary elements that demarcate transcriptional domains, ii) orchestrate DNA promoter and enhancer registration, and iii) associate with transcription regulators (Figure 1). Differentiating between cohesin functions likely depends both on the timing of its enrichment to DNA and post-translational modifications—the foremost of which appears composed of an acetylation code inscribed by Eco1/Ctf7/ESCO2 [4].

**Table 1:** Developmental and cytological phenotypes of cohesinopathies and potentially related maladies.

	RBS	CdLS	WABS	NBS	FA	DBA	TCS
*Developmental Phenotypes*							
Microcephaly	x	x	x	**x**	x	x	x
Craniofacial dysmorphia*	x	x	x	x	x	x	x
Cleft/arched palate	x	x	x	x		x	x
Cognitive retardation or decline	x	x	x	x	x		x
Growth retardation	x	x	x	x	x	x	
Syndactyly	x	x	x	x			x
Organ abnormalities**	x	x	x	x	x		x
Cardiac defects	x	x	x		x	x	
Limb reduction+	x	x		x	x		
Hearing loss		x	x		x		x
Skin pigmentation abnormalities			x	x	x		
Elevated Cancer Incidence				x	x	x	
Bone marrow/hematopoietic defects					x	x	
*Cytological Phenotypes*							
**Clastogen/genotoxin sensitivity**	x	x	x	**x**	x		
DNA breaks/translocations	x	x	x	x	x		
**HR/PCS** ++	**x**	ND	x				
Aneuploidy/Mitotic failure/Apoptosis	x	x			x	x	x
Multi/micronuclei	x	x			x		

Partial list of developmental and cytological effects in response to cohesion pathway mutations.

* Craniofacial dysmorphia include micrognathia, ear abnormalities, wide-set eyes, beaked or prominent nose, arched eyebrows, or low-set ears.

+ Limb reductions are often symmetric and involve all four limbs in RBS but predominant in upper extremities in CdLS. Limb reduction appears limited to the radius in NBS and FA.

** Organ abnormalities may include renal, urinary, gonadal, gastroesophageal, and others.

++ Detection of cryptic HR/PCS may require cell exposure to mitomycin. ND (Not Diagnostic): most studies document that HR/PCS is not elevated in CdLS cells [17], [18], [20], but see [48]. While HR/PCS is thus not efficacious as a diagnostic tool, numerous chromosomal aberrations are evident in CdLS cells upon exposure to genotoxic agents [20], revealing that CdLS cells may be predispositioned to PCS/HR. Bolded text represents examples of historical cytological diagnostic markers (HR/PCS for RBS, Clastogen sensitivity for FA). Phenotypes shown for potentially cohesinopathic-related developmental maladies (Ribosomopathies TCS and DBA, Nijmegen Breakage Disease, Fanconi Anemia—last four columns) that we speculate are similarly predicated on transcriptional dysregulation [1], [2], [5]–[8], [14], [26], [33], [41], [48], [55]–[57], [61], [63], [78], [79].

### *ESCO2* mutations result in elevated frequencies of apoptotic cells

Historically, the cataloging of childhood developmental disorders such as RBS was limited to physician-based descriptions. Included in RBS descriptions are cytological observations that included micronuclei, aneuploidy, and chromosomal abnormalities such as heterochromatin repulsion (HR)—often referred to as railroad track chromosomes or premature centromere separation (PCS). In humans, RBS arises through loss of both *ESCO2* gene copies [1]–[3]. *ESCO2* knockdown or mutation in model systems (including zebrafish, medaka, and mouse embryos) were found to recapitulate key RBS phenotypes [13]–[15]. In each case, elevated levels of apoptosis were observed in support of a model that mitotic failure induces apoptosis and further limits progenitor cell proliferation. *ESCO2* depletion from either mouse neuroepithelium or zebrafish embryos targeted brain and peripheral nervous system cells for death [13]–[15], formally suggesting a mechanism through which cognitive impairment could arise in RBS patients. In contrast, *ESCO2* depletion in a medaka model produced increased levels of apoptosis throughout the entire developing embryo [14]. Regardless, apoptotic loss and limited proliferation of progenitor cells remain popular mechanisms through which RBS developmental abnormalities arise, which span skeletal, organ, and cognitive defects.

### CdLS: A model of transcription dysregulation in cohesinopathies

Chromosome mis-segregation, aneuploidy, and the inability to induce apoptosis are features typically associated with tumor cells—rarely with cells from phenotypically pleiotropic and diverse developmental maladies. In this light, the model in which RBS arises through elevated apoptosis and limited cell proliferation may be exceptional. Thus, it is intriguing that CdLS, the sister cohesinopathy to RBS, arises through transcriptional dysregulation and not through mitotic failure or elevated levels of apoptosis [16]. For instance, cells from CdLS patients not only undergo normal mitosis, they contain chromosome structures overtly devoid of HR/PCS phenotypes [17]–[20]. CdLS is an autosomal dominant disease (*SMC1A* and *HDAC8* are X-linked) such that patients retain both a wildtype and dominant-mutant version of the cohesion gene [5]–[8], [12]. This heterozygosity likely accounts for the lack of PCS in CdLS patients, given that gene knockdowns (and likely homozygous mutations) of genes implicated in CdLS are either lethal or result in mitotic failure and apoptosis [21]–[23]. Based on early characterizations of NIPBL as a factor required for transcription regulation and cohesion [24], [25], numerous groups have linked cohesinopathic CdLS mutations to dysregulation of specific developmental and biochemical pathways. In genome-wide transcriptional microarrays of CdLS lymphoblastoid cell lines that harbor a mutation in *NIPBL*, 420 genes were differentially regulated, as compared to age and gender-matched controls. Interestingly, only modest levels of differential expression were reported (approximately 71% lower than 1.5-fold change), suggesting that CdLS phenotypes are caused by the accumulation of numerous yet small changes in expression—changes that may correlate with diminished cohesin binding near transcriptional start sites [26]. Mutations in *RAD21*, encoding another major subunit of the cohesin complex, result in dysregulation of the *APO* gene cluster—an effect also produced in *IGH* (Ig receptor genes), *IGF2-H19* (imprinted developmental genes), and *ESR1* (Estrogen receptor genes) [11], [27]–[32]. A proteomic regulation approach in CdLS cells mutated for either *SMC1A* or *SMC3* identified 46 proteins dysregulated to a fold change of 1.3 or greater, compared to age-gender-ethnicity controls. The functions of these dysregulated proteins are distributed throughout metabolism, cytoskeleton organization, protein fate, antioxidant detoxification, and RNA processing pathways. *In silico* network analysis established a link between these 46 dysregulated proteins and c-MYC—a transcription factor that, when mutated, plays critical roles in both cancer progression and aberrant development. Subsequent efforts confirmed *MYC* dysregulation in CdLS probands, similar to that previously reported in *RAD21* mutated cells, and that cohesin binding to the first exon of *c-MYC* is decreased in CdLS cells [31], [33].

### New evidence that ESCO2 is a critical regulator of both transcription and chromosome architecture

Do the more overt phenotypes of HR/PCS, mitotic failure, and elevated levels of apoptosis in RBS mask a role for *ESCO2* mutation in transcription dysregulation? In human cell lines, two recent reports document that ESCO proteins (ESCO1/EFO1 and ESCO2/EFO2 are both acetyltransferases that target SMC3 [34]–[38]) impact transcriptional regulation. ESCO2 associates with CoREST transcriptional repressor complex subunits and methyltransferases (SETDB1, G9a, and suv39h1) and demethylases (LSD1), which repress transcription through H3 modifications [39]. Knockdown of either methyltransferase SETDB1 or suv39h1or demethylase LSD1 relieved ESCO2-dependent transcription repression, suggesting that ESCO2 recruits chromatin modifiers to affect repression. Consistent with this possibility, repression was unaffected by mutation within the ESCO2 acetyltransferase domain [39]. Similar findings involving repression by LSD1 and binding of chromatin modifiers (LSD1, SETDB1, G9a, and suv39h1) were reported for ESCO1 and also in an acetyltransferase-independent fashion [40]. Thus, the ESCO/EFO family exhibits numerous functions: as an acetyltransferase that modifies cohesin (and likely other substrates) and as a scaffold through which chromatin modifiers are recruited.

Hints regarding ESCO2 targets recently were identified in human, mouse neuron, and teleost medaka models. In addition to chromatin remodeling complexes (CoREST, LSD1, HDACs), ESCO2 binds to Notch from human cell extracts [41]. Notch signaling is a major regulator of developmental programs—including organ and brain development. *ESCO2* expression suppressed Notch-dependent activation, in a manner independent of ESCO2 acetyltransferase activity. Instead, ESCO2 binds directly to the Notch Intracellular Domain (NICD), a domain released upon receptor activation/cleavage, and prevents NICD activation of the transcription regulator CBF1 involved in neural progenitor cell proliferation and differentiation. ESCO2 depletion in mice inhibited neural cell differentiation (shorter neurites), while overexpression resulted in cells that exhibited longer neurites [41]. In medaka fish models, diminished ESCO2 function resulted in decreased expression of both Notch1a and Notch3 [14]. While Notch1a is a potent regulator of neuronal cell proliferation and head development, Notch3 is a vascular differentiation marker and previously linked to heart malformations in the medaka model. These studies potentially link mutated *ESCO2* RBS manifestations such as microcephaly, cognitive retardation, and heart defects to transcriptional dysregulation of Notch pathways [14], [41].

ESCO2-deployment of transcriptional programs may not be surprising given early evidence that ESCO family members are critical regulators of chromatin architecture. The earliest study of the ESCO2 homolog in yeast (Eco1/Ctf7) revealed that mutations produced both cohesion and chromosome condensation defects—effects mirrored in both yeast and vertebrate cell cohesion mutations [14], [15], [42]–[45]. In zebrafish, microarray analysis confirmed alteration of gene transcripts in tissues depleted of ESCO2, a population of which overlapped with those altered in cohesin mutation [13]. Intriguingly, this shared subset of genes was dysregulated in opposition: genes repressed upon ESCO2 depletion were upregulated in cohesin mutation and vice versa. When viewed through the lens of ESCO2 as both a cohesin acetyltransferase and chromatin-remodeler scaffold, and that cohesin acetylation is read as a code through which different cell processes differentially respond [4], this apparent discrepancy is easily accommodated and suggests that transcriptional changes in either direction can alter normal development.

### Overlap of cell phenotypes supports a unifying transcription-based cohesinopathy model

If our RBS transcription dysregulation model is correct, then transcriptional dysregulation in RBS cells should map to a subset of dysregulated genes in CdLS. This prediction proves true. Microarray analyses of CdLS proband patient cells identified approximately ten dysregulated genes that both accurately distinguished between controls and CdLS probands and correlated with CdLS severity. When this same gene set was tested against two RBS probands, the RBS samples were included in the CdLS group, revealing in concept a transcriptional match between CdLS and RBS gene dysregulations [26]. Is the converse relationship true: do CdLS cells exhibit elevated levels of mitotic failure and apoptosis? On the one hand, CdLS is not a recessive condition, but instead is autosomal dominant. Thus, CdLS patient cells typically retain one normal cohesion gene homolog and thus do not manifest the mitotic failure or limited cell proliferation observed in RBS patient cells in which both gene homologs are altered. However, experiments from yeast, man, and fish reveal the essential nature of these genes and that elevated levels of apoptosis can occur in response to either mutation or knockdown of *SMC1A*, *RAD21*, *SMC3*, or *PDS5* [4], [10], [21], [23], [33], [46]–[49]. *RAD21* is particularly intriguing given that caspase-dependent cleavage of RAD21 produces a C-terminal product that further promotes the apoptotic response pathway [50]. Clinical implications are intriguing as well: knockdown of *RAD21* in human breast cancer or *SMC1A* in adenocarcinoma cells both elicited an apoptotic-type response [23], [51]. Thus, gene mutations causative for RBS and CdLS have the capacity to exhibit a sliding scale of chromosome segregation defects, elevated levels of apoptosis, and reduced cell proliferation that are superimposed on top of transcription dysregulation—the latter of which we speculate is causatively associated with developmental defects. Given that mutation of ESCO2 acetyltransferase results in RBS developmental abnormalities, might mutations in an opposing activity similarly be of clinical interest? HDAC8 (Hos1 in yeast) is a de-acetylase that opposes ESCO2 modification of SMC3 [12], [52]–[54]. Recent studies reveal that *HDAC8* mutation results in transcription dysregulation—similar to *NIPBL* mutants in CdLS—and decreased cohesin occupancy of localization sites. Intriguingly, cohesins remain bound to chromatin in HDAC8 deficient cells, even after mitosis, which may explain the delay in anaphase and mitotic failure in these cells through which RBS is also phenocopied [12].

### Cohesinopathies may not be so rare after all

The range of cohesinopathies continues to expand, projecting that the number of ESCO2-dependent maladies will increase significantly as molecular genetics continue to link mutations in *ESCO2* (or *ESCO1*) to other multi-spectrum disorders. For instance, the majority of RBS patients exhibit significant cognitive impairment, making it difficult to exclude allele-specific *ESCO2* mutations as a contributing factor in any number of cognitive syndromes. Moreover, cells from RBS patients proved indistinguishable from those of Fanconi Anemia (FA) when scored using an FA-specific diagnostic chromosome breakage test [55]. The same diagnostic assay also positively identified cells from Warsaw Breakage Syndrome (WABS) patients as FA-like. WABS presents as a multispectrum developmental malady that arises from mutations within the DNA helicase DDX11/ChlR1 [56], [57]. Chl1, the yeast homolog of ChlR1/DDX11, associates with Eco1/Ctf7 (ESCO2), and mutations in either yeast or human homolog produces significant cohesion defects [4]. Further blurring the lines between developmental maladies is the recent finding that Chl1 is critical for Scc2 (NIPBL) recruitment to DNA—conceptually linking WABS, CdLS, and RBS [58].

Genotoxic sensitivity appears to be another theme that runs throughout cohesinopathic syndromes. For instance, cells from patients afflicted with FA, WABS, CdLS, or RBS all exhibit genotoxic sensitivities [1], [3], [17], [20], [23], [55], [59], [60]. Intriguingly, all cohesinopathic cells tested to date also produce HR/PCS or other chromosomal aberrations when exposed to mitomycin [56]. Are genotoxic sensitivities a critical etiologic agent in developmental abnormalities? CdLS may be particularly instructive in that these cells exhibit some deficiencies in DNA repair, but deficiencies that are relatively limited in scope. For instance, a study that included seven CdLS patient cell lines failed to identify any that were UV sensitive, and less than half exhibited X-irradiation sensitivity (and then only at very high exposure levels). Conversely, all CdLS tested exhibit MMC sensitive [20]. A similar range of genotoxic sensitivities occurs in RBS cells [11], [61]. Thus, genotoxic sensitivities remain a useful diagnostic tool but likely are not uniquely etiologic in nature. RBS cells also exhibit DNA replication defects, but this phenotype has yet to be linked to other cohesinopathies, and the causality of this phenotype is complicated by numerous findings that Eco1/Ctf7/ESCO2 function is tightly coordinated to DNA replication fork components [4]. Intriguingly, cohesin roles during DNA damage repair change based on the proximity to the break site: break-proximal cohesion promotes DNA repair while global cohesion promotes high fidelity chromosome segregation [59]. The mechanism through which cohesins promote DNA repair and the extent that cohesinopathic genotoxic sensitivities contribute to developmental maladies remains an open question. Instead, we posit that many of the presenting manifestations (beyond developmental abnormalities) likely arise through additional roles played by those factors—a model supported by findings that DNA repair, condensation, and cohesion are differentially sensitive to changes in cohesin gene dosages including *SCC2/NIPBL*, *MCD1/RAD21* and *PDS5B* [19]. In Nijmegen Breakage Syndrome (NBS) and FA patient cells, for instance, DNA damaging sensing and repair enzymes themselves are defective [62], [63]; it is the additional loss of these activities that likely correlate to the added complexities of cancers and/or anemia.

Cohesinopathies, defined here as transcription dysregulation disorders, likely encompass other maladies such as ribosomopathies (Table 1). Ribosomopathies include Diamond Blackfan anemia (DBA) and Treacher-Collins syndrome (TCS). In the case of DBA, the presenting hematologic abnormality is anemia, but this does not exclude transcription dysregulation as the basis for developmental defects also present in DBA patients. TCS, on the other hand, is distinguished primarily by craniofacial irregularities that are similar to those of cohesinopathies [64]–[66]. How are cohesinopathies and ribosomopathies linked? Early studies of Eco1/Ctf7 and cohesin function in rDNA architecture [42], [43] were quickly followed by reports of cohesin localization to rDNA and function in rDNA segregation, recombination and maintenance [67]–[71]. CdLS mouse cell transcriptomes revealed a vast array of gene dysregulations previously shown to correlate with developmental phenotypes [72], including *TCOF1*, which is required for ribosome biogenesis and in which mutations result in severe facial/cranial dysmorphia [73]. Even transient cohesin inactivation in yeast, and specifically during G1 prior to its role in chromosome segregation, results in transcription dyregulation—the largest class comprising ribosome biogenesis/maturation. Based on these findings, the first formal statement linking ribosomopathies (Treacher-Collins Syndrome and Diamond Blackfan anemia) to cohesinopathies was articulated [74]. More recent evidence reveals that mutation in either Ctf7/Eco1 (ESCO2) or cohesin significantly reduces both rDNA transcription and ribosome subunit productions, leading to translation initiation defects and decreased protein synthesis [74], [75]. Further support emanates from human cell studies in that ESCO2 localizes to nucleoli, a heterochromatic domain required for rRNA production [15], [61]. In human cohesinopathic cells, regulation of MYC, p53, and MDM2 are all affected by ribosome biogenesis [75]. Thus, it is tempting to speculate that cohesinopathies represent an umbrella under which ribosomopathies reside ([76] for an excellent review). In this light, testing for rRNA maturation/ribosome biogenesis defects in the roughly 35% of genetically uncharacterized CdLS patients may be of value [74], [77]. Moreover, we look forward to rigorous testing of our speculative model that many developmental maladies (RBS, WABS, NBS, FA, DBA, and TCS), currently posited as unique in etiology, may be based on transcriptional dysregulation maladies akin to CdLS, with additional levels of complexity.
